# *cis*,*trans*,*cis*,*cis*-7-*tert*-Butyl­dimethyl­silyl­oxy-4,10-dimethyl­tetra­cyclo[5.4.1.0^4,12^.0^10,12^]dodecan-2-one

**DOI:** 10.1107/S1600536810000887

**Published:** 2010-01-13

**Authors:** Philipp Weyermann, Reinhart Keese, Helen Stoeckli-Evans

**Affiliations:** a Department of Chemistry and Biochemistry, University Bern, Freiestrasse 3, CH-3012 Bern, Switzerland; b Institute of Physics, University of Neuchâtel, rue Emile-Argand 11, CH-2009 Neuchâtel, Switzerland

## Abstract

In the structure of the title compound, C_20_H_34_O_2_Si, a *cis*,*trans*,*cis*,*cis*-[4.5.5.5]fenestrane derivative, the geometry of the central C(C)_4_ substructure shows considerable distortion from an ideal tetra­hedral arrangement towards planarity, with two opposite bridgehead bond angles of 128.87 (18) and 122.83 (17)°. The other bridgehead angle of the *trans*-bicyclo­[3.3.0]octane subunit is also large [126.57 (19)°].

## Related literature

For the synthesis and structures of related compounds, see: Thommen *et al.* (1996 ▶); Wang *et al.* (1996 ▶); Weyermann (1997 ▶); Weyermann & Keese (2010 ▶). For information on planarizing distortions in the central C(C)_4_ moiety, see: Keese (2006 ▶). For methods to enhance the planarizing distortions in the central C(C)_4_ substructure, see: Luef & Keese (1993 ▶). For an analysis of the bond angles and other details concerning *trans*-fused bicyclo­[3.3.0]octa­nes, see: Hirschi *et al.* (1992 ▶). For information concerning the Pauson–Khand reaction, see: Khand, Knox, Pauson & Watts (1973 ▶); Khand, Knox, Pauson, Watts & Foreman (1973 ▶).
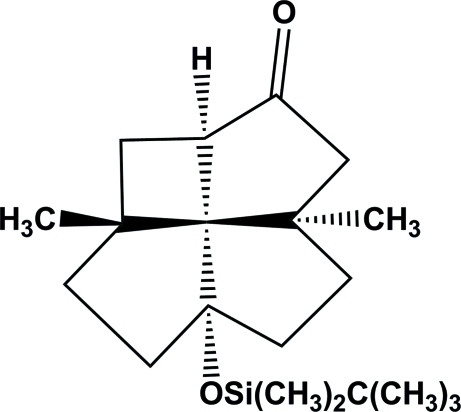


## Experimental

### 

#### Crystal data

C_20_H_34_O_2_Si
*M* *_r_* = 334.56
Orthorhombic, 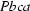

*a* = 13.7374 (13) Å
*b* = 14.7647 (11) Å
*c* = 19.2829 (12) Å
*V* = 3911.1 (5) Å^3^
*Z* = 8
Mo *K*α radiation
μ = 0.13 mm^−1^
*T* = 223 K
0.53 × 0.42 × 0.34 mm

#### Data collection

Stoe AED2 four-circle diffractometer
7284 measured reflections
3642 independent reflections
2718 reflections with *I* > 2σ(*I*)
*R*_int_ = 0.042
3 standard reflections every 60 min  intensity decay: <1%

#### Refinement

*R*[*F* ^2^ > 2σ(*F* ^2^)] = 0.048
*wR*(*F* ^2^) = 0.116
*S* = 1.07
3642 reflections
216 parameters
H-atom parameters constrained
Δρ_max_ = 0.28 e Å^−3^
Δρ_min_ = −0.29 e Å^−3^

### 

Data collection: *STADI-4* (Stoe & Cie, 1997 ▶); cell refinement: *STADI-4*; data reduction: *X-RED* (Stoe & Cie, 1997 ▶); program(s) used to solve structure: *SHELXS97* (Sheldrick, 2008 ▶); program(s) used to refine structure: *SHELXL97* (Sheldrick, 2008 ▶); molecular graphics: *PLATON* (Spek, 2009 ▶); software used to prepare material for publication: *SHELXL97*.

## Supplementary Material

Crystal structure: contains datablocks I, global. DOI: 10.1107/S1600536810000887/fk2011sup1.cif

Structure factors: contains datablocks I. DOI: 10.1107/S1600536810000887/fk2011Isup2.hkl

Additional supplementary materials:  crystallographic information; 3D view; checkCIF report

## Figures and Tables

**Fig. 1.**
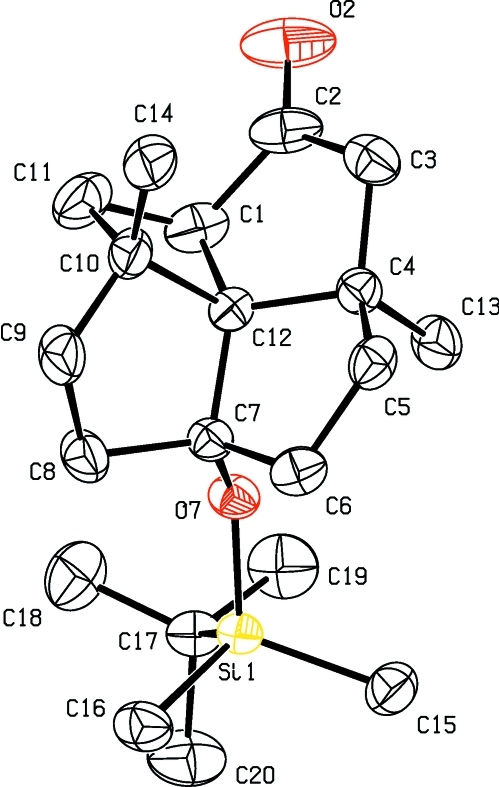
The molecular structure of compound (3), with displacement ellipsoids drawn at the 50% probability level [the H-atoms have been omitted for clarity].

**Fig. 2.**
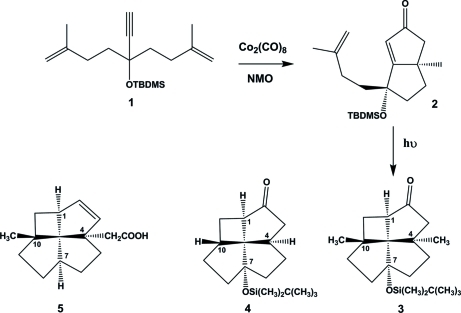
The synthesis of the title compound.

## supplementary crystallographic information

### Comment

Fenestranes are a unique class of hydrocarbons and contain a quaternary C atom in the center of the tetracyclic structure. These compounds are of interest for the planarizing distortions in the central C(C)_4_ moiety, apparent in two opposite bond angles larger than the bond angle of 109.47° in a regular tetrahedral arrangement (Keese, 2006). Systematic investigations of the structural features in a variety of such molecules by semiempirical methods have revealed that they can be enhanced by ring contraction, inversion at one (or more) of the four bridgehead centers, giving rise to a *trans*-fused bicylo[3.3.0]octane subunit, by introduction of a bridgehead double bond (Luef & Keese, 1993) and by alkyl groups at the peripheral bridgehead positions. As part of our efforts to prepare fenestranes with a combination of structural features for enhanced planarizing distortions we have prepared the title compound, (3), from the yne-diene (1) by a Co_2_(CO)_8_-induced cyclocarbonylation reaction (Pauson-Khand reaction - Khand *et al.*, 1973*a*, 1973*b*) followed by a photoinduced intramolecular olefin-enone *cyclo*-addition of (2) [see Scheme 2] (Weyermann, 1997; Weyermann & Keese, 2010).

The molecular structure of the title compound (3) is illustrated in Fig. 1, and geometrical parameters are given in the Supplementary information and the archived CIF. In (3) the bridgehead bond angles C1—C12—C7 and C4—C12—C10 are 128.87 (18)° and 122.83 (17)°, respectively. In comparison in compound (4), the *cis,trans,cis,cis* [4.5.5.5]fenestrane without the methyl groups at the bridgehead positions C4 and C10 (Thommen *et al.*, 1996), the same bridgehead bond angles are 131.1 (2)° and 120.2 (2)°, respectively. In the related *cis,trans,cis,cis*[4.5.5.5.]fenestrene (5), bearing only one bridgehead substituent at C4, the bridgehead bond angles are slightly different to those in (3) and (4); C1—C12—C7 and C4—C12—C10 are 134.9 (2)° and 119.2 (2)°, respectively (Wang *et al.*,  1996).

An earlier analysis of the bond angles in *trans*-fused bicyclo[3.3.0]octanes (Hirschi *et al.*,  1992) revealed that the bond angles at the bridgehead centres are always larger than the normal tetrahedral angle. In line with these findings in (3) bond angle C3—C4—C5 is 126.57 (19)°, and 127.0 (2)° in (4).

Salient features in (3) are the bond distances and angles involving the methyl substituents C13 and C14. Bonds C4—C13 and C10—C14 are 1.548 (3) and 1.526 (3) Å, respectively, while bond angles C12—C4—C13 and C12—C10—C14 are 111.99 (18) and 124.53 (18)°, respectively. The torsional angles C13—C4—C12—C10 and C14—C10—C12—C4 are -173.39 (19) and 9.9 (3)°, respectively, indicating that the deviation from a strictly ecliptic orientation is rather small.

In conclusion it can be seen that the introduction of the methyl substituents in (3) hardly enhances the planoid distortions in the central C(C)_4_ substructure. Apparently accumulation of three quaternary C-atoms, adjacent to one another in the tetracyclic fenestrane (3), leads to a different adjustment of the steric interactions.

### Experimental

The synthesis of the title compound, (3), is illustrated in Fig. 2. 2,8-dimethyl-5-ethinyl-5-(*tert*.-butyldimethylsilyloxy =OTBDMS)-1,8-nonadiene (1) was treated with 1.15 molequivalent of Co_2_(CO)_8_ in tetrahydrofuran/CH_2_Cl_2_ (1:1) and *N*-methylmorpholine-N oxide (NMO) at r.t. to give the enone (2), together with another diastereomer. Irradiation with UV light (254 nm) gave the title fenestranone (3) in 79% isolated yield. Colourless needle-like crystals of (3) were obtained by crystallization from hexane at 253 K (m.p. 356–348 K).

### Refinement

The H-atoms were included in calculated positions and treated as riding atoms: C—H = 0.97 - 0.99 Å with *U*_iso_(H) = k × *U*_eq_(C), where k = 1.2 for CH and CH_2_ H-atoms and 1.5 for methyl H-atoms.

### Crystal data

**Table:**

C_20_H_34_O_2_Si	*F*(000) = 1472
*M**_r_* = 334.56	*D*_x_ = 1.136 Mg m^−^^3^
Orthorhombic, *P**b**c**a*	Mo *K*α radiation, λ = 0.71073 Å
Hall symbol: -P 2ac 2ab	Cell parameters from 20 reflections
*a* = 13.7374 (13) Å	θ = 14–17.7°
*b* = 14.7647 (11) Å	µ = 0.13 mm^−^^1^
*c* = 19.2829 (12) Å	*T* = 223 K
*V* = 3911.1 (5) Å^3^	Block, colourless
*Z* = 8	0.53 × 0.42 × 0.34 mm

### Data collection

**Table:**

Stoe AED2 four-circle diffractometer	*R*_int_ = 0.042
Radiation source: fine-focus sealed tube	θ_max_ = 25.5°, θ_min_ = 2.1°
graphite	*h* = −16→16
2θ/ω scans	*k* = 0→17
7284 measured reflections	*l* = 0→23
3642 independent reflections	3 standard reflections every 60 min
2718 reflections with *I* > 2σ(*I*)	intensity decay: <1%

### Refinement

**Table:**

Refinement on *F*^2^	Secondary atom site location: difference Fourier map
Least-squares matrix: full	Hydrogen site location: inferred from neighbouring sites
*R*[*F*^2^ > 2σ(*F*^2^)] = 0.048	H-atom parameters constrained
*wR*(*F*^2^) = 0.116	*w* = 1/[σ^2^(*F*_o_^2^) + (0.0378*P*)^2^ + 2.3403*P*] where *P* = (*F*_o_^2^ + 2*F*_c_^2^)/3
*S* = 1.07	(Δ/σ)_max_ < 0.001
3642 reflections	Δρ_max_ = 0.28 e Å^−^^3^
216 parameters	Δρ_min_ = −0.29 e Å^−^^3^
0 restraints	Extinction correction: *SHELXL97* (Sheldrick, 2008), Fc^*^=kFc[1+0.001xFc^2^λ^3^/sin(2θ)]^-1/4^
Primary atom site location: structure-invariant direct methods	Extinction coefficient: 0.0018 (5)

### Special details

**Table:**

Geometry. Bond distances, angles *etc*. have been calculated using the rounded fractional coordinates. All su's are estimated from the variances of the (full) variance-covariance matrix. The cell e.s.d.'s are taken into account in the estimation of distances, angles and torsion angles
Refinement. Refinement of *F*^2^ against ALL reflections. The weighted *R*-factor *wR* and goodness of fit *S* are based on *F*^2^, conventional *R*-factors *R* are based on *F*, with *F* set to zero for negative *F*^2^. The threshold expression of *F*^2^ > σ(*F*^2^) is used only for calculating *R*-factors(gt) *etc*. and is not relevant to the choice of reflections for refinement. *R*-factors based on *F*^2^ are statistically about twice as large as those based on *F*, and *R*- factors based on ALL data will be even larger.

### Fractional atomic coordinates and isotropic or equivalent isotropic displacement parameters (Å^2^)

**Table:**

	*x*	*y*	*z*	*U*_iso_*/*U*_eq_	
Si1	0.18635 (4)	0.81357 (4)	0.04887 (3)	0.0263 (2)	
O2	0.27615 (14)	0.95616 (16)	0.38174 (11)	0.0711 (8)	
O7	0.17598 (10)	0.86338 (10)	0.12503 (7)	0.0312 (5)	
C1	0.21973 (17)	0.97101 (17)	0.26241 (13)	0.0408 (8)	
C2	0.21585 (18)	0.93807 (18)	0.33818 (13)	0.0454 (9)	
C3	0.12597 (18)	0.87879 (17)	0.35050 (12)	0.0401 (8)	
C4	0.09843 (16)	0.84971 (14)	0.27688 (11)	0.0301 (7)	
C5	−0.00409 (17)	0.83122 (15)	0.24976 (12)	0.0350 (7)	
C6	0.00658 (16)	0.84850 (16)	0.17076 (12)	0.0355 (7)	
C7	0.09821 (15)	0.90983 (14)	0.15884 (11)	0.0274 (6)	
C8	0.0748 (2)	0.99911 (16)	0.12245 (12)	0.0427 (8)	
C9	0.0247 (2)	1.05514 (15)	0.17828 (12)	0.0457 (9)	
C10	0.07572 (17)	1.03098 (14)	0.24635 (12)	0.0346 (7)	
C11	0.1814 (2)	1.06806 (17)	0.25435 (15)	0.0527 (9)	
C12	0.12488 (15)	0.93286 (14)	0.23397 (10)	0.0264 (6)	
C13	0.16461 (18)	0.76782 (15)	0.25992 (13)	0.0403 (8)	
C14	0.0109 (2)	1.05498 (16)	0.30795 (13)	0.0445 (8)	
C15	0.15738 (19)	0.68993 (15)	0.05640 (13)	0.0419 (8)	
C16	0.10610 (18)	0.86511 (17)	−0.01811 (12)	0.0418 (8)	
C17	0.31834 (16)	0.82806 (16)	0.02537 (12)	0.0340 (7)	
C18	0.3464 (2)	0.92884 (18)	0.02703 (16)	0.0554 (10)	
C19	0.38166 (19)	0.7765 (2)	0.07777 (15)	0.0543 (10)	
C20	0.3373 (2)	0.7910 (2)	−0.04778 (14)	0.0584 (10)	
H1	0.27950	0.95590	0.23620	0.0490*	
H3A	0.14170	0.82630	0.37960	0.0480*	
H3B	0.07340	0.91340	0.37240	0.0480*	
H5A	−0.05160	0.87270	0.27060	0.0420*	
H5B	−0.02390	0.76870	0.25910	0.0420*	
H6A	−0.05180	0.87870	0.15290	0.0430*	
H6B	0.01440	0.79090	0.14620	0.0430*	
H8A	0.13430	1.02890	0.10630	0.0510*	
H8B	0.03140	0.98920	0.08280	0.0510*	
H9A	−0.04470	1.04020	0.18070	0.0550*	
H9B	0.03140	1.12000	0.16840	0.0550*	
H11A	0.20580	1.09900	0.21290	0.0630*	
H11B	0.19070	1.10560	0.29570	0.0630*	
H13A	0.23230	0.78530	0.26520	0.0600*	
H13B	0.15310	0.74850	0.21250	0.0600*	
H13C	0.14990	0.71840	0.29140	0.0600*	
H14A	0.04770	1.04860	0.35060	0.0670*	
H14B	−0.04470	1.01460	0.30900	0.0670*	
H14C	−0.01140	1.11700	0.30340	0.0670*	
H15A	0.19140	0.66470	0.09600	0.0630*	
H15B	0.17790	0.65890	0.01450	0.0630*	
H15C	0.08780	0.68220	0.06260	0.0630*	
H16A	0.03900	0.86290	−0.00250	0.0630*	
H16B	0.11240	0.83170	−0.06120	0.0630*	
H16C	0.12510	0.92760	−0.02560	0.0630*	
H18A	0.30640	0.96200	−0.00570	0.0830*	
H18B	0.41440	0.93540	0.01460	0.0830*	
H18C	0.33600	0.95270	0.07330	0.0830*	
H19A	0.36730	0.79770	0.12430	0.0810*	
H19B	0.44990	0.78700	0.06750	0.0810*	
H19C	0.36780	0.71220	0.07460	0.0810*	
H20A	0.29860	0.82450	−0.08110	0.0880*	
H20B	0.31960	0.72740	−0.04950	0.0880*	
H20C	0.40580	0.79760	−0.05900	0.0880*	

### Atomic displacement parameters (Å^2^)

**Table:**

	*U*^11^	*U*^22^	*U*^33^	*U*^12^	*U*^13^	*U*^23^
Si1	0.0267 (3)	0.0288 (3)	0.0234 (3)	0.0009 (3)	−0.0002 (2)	−0.0016 (2)
O2	0.0468 (12)	0.1080 (18)	0.0584 (13)	0.0074 (12)	−0.0202 (10)	−0.0360 (12)
O7	0.0315 (8)	0.0354 (8)	0.0266 (8)	0.0075 (7)	0.0024 (6)	−0.0031 (6)
C1	0.0288 (12)	0.0511 (15)	0.0425 (14)	−0.0097 (11)	0.0079 (10)	−0.0142 (12)
C2	0.0339 (13)	0.0608 (17)	0.0415 (14)	0.0103 (12)	−0.0048 (11)	−0.0216 (13)
C3	0.0426 (14)	0.0460 (14)	0.0318 (13)	0.0112 (12)	−0.0006 (11)	0.0006 (11)
C4	0.0323 (12)	0.0287 (11)	0.0292 (11)	0.0019 (10)	0.0013 (9)	0.0004 (9)
C5	0.0323 (12)	0.0314 (12)	0.0414 (12)	−0.0064 (10)	0.0077 (10)	−0.0011 (10)
C6	0.0271 (12)	0.0399 (13)	0.0394 (13)	0.0012 (10)	−0.0039 (10)	−0.0063 (11)
C7	0.0284 (11)	0.0281 (11)	0.0256 (11)	0.0048 (9)	0.0022 (9)	−0.0019 (9)
C8	0.0627 (17)	0.0343 (13)	0.0310 (12)	0.0148 (12)	0.0064 (12)	0.0048 (10)
C9	0.0645 (17)	0.0298 (13)	0.0429 (14)	0.0164 (12)	0.0083 (13)	0.0055 (11)
C10	0.0435 (14)	0.0248 (11)	0.0355 (12)	−0.0003 (10)	0.0094 (11)	−0.0008 (10)
C11	0.0632 (18)	0.0436 (14)	0.0512 (15)	−0.0225 (14)	0.0151 (14)	−0.0114 (13)
C12	0.0238 (10)	0.0266 (10)	0.0287 (11)	−0.0010 (9)	0.0032 (9)	−0.0028 (9)
C13	0.0480 (15)	0.0355 (13)	0.0375 (14)	0.0132 (11)	−0.0011 (11)	0.0034 (11)
C14	0.0577 (16)	0.0332 (13)	0.0427 (14)	0.0102 (12)	0.0110 (13)	−0.0047 (11)
C15	0.0503 (15)	0.0333 (12)	0.0421 (14)	−0.0037 (11)	0.0075 (12)	−0.0053 (11)
C16	0.0405 (14)	0.0484 (15)	0.0365 (13)	0.0056 (12)	−0.0095 (11)	−0.0016 (11)
C17	0.0288 (11)	0.0417 (13)	0.0314 (11)	0.0007 (10)	−0.0003 (10)	−0.0056 (10)
C18	0.0435 (15)	0.0518 (17)	0.0710 (19)	−0.0147 (13)	0.0071 (14)	−0.0009 (15)
C19	0.0355 (14)	0.0663 (19)	0.0610 (17)	0.0126 (13)	−0.0082 (13)	−0.0025 (15)
C20	0.0459 (16)	0.085 (2)	0.0443 (15)	−0.0068 (14)	0.0161 (13)	−0.0147 (15)

### Geometric parameters (Å, °)

**Table:**

Si1—O7	1.6486 (15)	C5—H5B	0.9800
Si1—C15	1.874 (2)	C6—H6A	0.9800
Si1—C16	1.861 (2)	C6—H6B	0.9800
Si1—C17	1.881 (2)	C8—H8A	0.9800
O2—C2	1.210 (3)	C8—H8B	0.9800
O7—C7	1.427 (3)	C9—H9A	0.9800
C1—C2	1.541 (4)	C9—H9B	0.9800
C1—C11	1.535 (4)	C11—H11A	0.9800
C1—C12	1.522 (3)	C11—H11B	0.9800
C2—C3	1.532 (4)	C13—H13A	0.9700
C3—C4	1.531 (3)	C13—H13B	0.9700
C4—C5	1.527 (3)	C13—H13C	0.9700
C4—C12	1.524 (3)	C14—H14A	0.9700
C4—C13	1.548 (3)	C14—H14B	0.9700
C5—C6	1.552 (3)	C14—H14C	0.9700
C6—C7	1.568 (3)	C15—H15A	0.9700
C7—C8	1.528 (3)	C15—H15B	0.9700
C7—C12	1.533 (3)	C15—H15C	0.9700
C8—C9	1.522 (3)	C16—H16A	0.9700
C9—C10	1.530 (3)	C16—H16B	0.9700
C10—C11	1.559 (4)	C16—H16C	0.9700
C10—C12	1.616 (3)	C18—H18A	0.9700
C10—C14	1.526 (3)	C18—H18B	0.9700
C17—C18	1.537 (4)	C18—H18C	0.9700
C17—C19	1.535 (4)	C19—H19A	0.9700
C17—C20	1.535 (4)	C19—H19B	0.9700
C1—H1	0.9900	C19—H19C	0.9700
C3—H3A	0.9800	C20—H20A	0.9700
C3—H3B	0.9800	C20—H20B	0.9700
C5—H5A	0.9800	C20—H20C	0.9700
			
O7—Si1—C15	110.32 (10)	C5—C6—H6A	110.00
O7—Si1—C16	112.62 (9)	C5—C6—H6B	110.00
O7—Si1—C17	104.31 (9)	C7—C6—H6A	110.00
C15—Si1—C16	109.05 (11)	C7—C6—H6B	110.00
C15—Si1—C17	109.52 (11)	H6A—C6—H6B	108.00
C16—Si1—C17	110.94 (11)	C7—C8—H8A	111.00
Si1—O7—C7	133.31 (13)	C7—C8—H8B	111.00
C2—C1—C11	112.3 (2)	C9—C8—H8A	111.00
C2—C1—C12	101.26 (18)	C9—C8—H8B	111.00
C11—C1—C12	90.88 (18)	H8A—C8—H8B	109.00
O2—C2—C1	124.4 (2)	C8—C9—H9A	111.00
O2—C2—C3	124.8 (2)	C8—C9—H9B	111.00
C1—C2—C3	110.8 (2)	C10—C9—H9A	111.00
C2—C3—C4	102.45 (18)	C10—C9—H9B	111.00
C3—C4—C5	126.57 (19)	H9A—C9—H9B	109.00
C3—C4—C12	102.63 (17)	C1—C11—H11A	114.00
C3—C4—C13	105.66 (18)	C1—C11—H11B	114.00
C5—C4—C12	100.25 (17)	C10—C11—H11A	114.00
C5—C4—C13	109.25 (18)	C10—C11—H11B	114.00
C12—C4—C13	111.99 (18)	H11A—C11—H11B	111.00
C4—C5—C6	102.69 (18)	C4—C13—H13A	109.00
C5—C6—C7	108.35 (18)	C4—C13—H13B	109.00
O7—C7—C6	112.99 (17)	C4—C13—H13C	109.00
O7—C7—C8	111.25 (17)	H13A—C13—H13B	109.00
O7—C7—C12	111.07 (16)	H13A—C13—H13C	110.00
C6—C7—C8	113.38 (18)	H13B—C13—H13C	110.00
C6—C7—C12	100.46 (17)	C10—C14—H14A	109.00
C8—C7—C12	107.04 (17)	C10—C14—H14B	109.00
C7—C8—C9	103.85 (18)	C10—C14—H14C	109.00
C8—C9—C10	105.8 (2)	H14A—C14—H14B	109.00
C9—C10—C11	115.4 (2)	H14A—C14—H14C	109.00
C9—C10—C12	105.88 (17)	H14B—C14—H14C	110.00
C9—C10—C14	110.3 (2)	Si1—C15—H15A	109.00
C11—C10—C12	86.58 (16)	Si1—C15—H15B	109.00
C11—C10—C14	112.6 (2)	Si1—C15—H15C	109.00
C12—C10—C14	124.53 (18)	H15A—C15—H15B	110.00
C1—C11—C10	90.09 (18)	H15A—C15—H15C	109.00
C1—C12—C4	107.85 (17)	H15B—C15—H15C	109.00
C1—C12—C7	128.87 (18)	Si1—C16—H16A	109.00
C1—C12—C10	88.44 (16)	Si1—C16—H16B	109.00
C4—C12—C7	106.11 (17)	Si1—C16—H16C	109.00
C4—C12—C10	122.83 (17)	H16A—C16—H16B	109.00
C7—C12—C10	103.81 (16)	H16A—C16—H16C	110.00
Si1—C17—C18	110.29 (16)	H16B—C16—H16C	109.00
Si1—C17—C19	109.34 (16)	C17—C18—H18A	109.00
Si1—C17—C20	110.14 (16)	C17—C18—H18B	109.00
C18—C17—C19	108.9 (2)	C17—C18—H18C	109.00
C18—C17—C20	108.8 (2)	H18A—C18—H18B	110.00
C19—C17—C20	109.4 (2)	H18A—C18—H18C	109.00
C2—C1—H1	116.00	H18B—C18—H18C	109.00
C11—C1—H1	116.00	C17—C19—H19A	109.00
C12—C1—H1	116.00	C17—C19—H19B	109.00
C2—C3—H3A	111.00	C17—C19—H19C	109.00
C2—C3—H3B	111.00	H19A—C19—H19B	109.00
C4—C3—H3A	111.00	H19A—C19—H19C	110.00
C4—C3—H3B	111.00	H19B—C19—H19C	109.00
H3A—C3—H3B	109.00	C17—C20—H20A	110.00
C4—C5—H5A	111.00	C17—C20—H20B	109.00
C4—C5—H5B	111.00	C17—C20—H20C	109.00
C6—C5—H5A	111.00	H20A—C20—H20B	109.00
C6—C5—H5B	111.00	H20A—C20—H20C	109.00
H5A—C5—H5B	109.00	H20B—C20—H20C	109.00
			
C15—Si1—O7—C7	−92.99 (19)	C5—C4—C12—C1	170.87 (17)
C16—Si1—O7—C7	29.1 (2)	C5—C4—C12—C7	−47.9 (2)
C17—Si1—O7—C7	149.49 (18)	C5—C4—C12—C10	70.9 (2)
O7—Si1—C17—C18	−55.54 (19)	C13—C4—C12—C1	−73.4 (2)
O7—Si1—C17—C19	64.20 (18)	C13—C4—C12—C7	67.8 (2)
O7—Si1—C17—C20	−175.58 (16)	C13—C4—C12—C10	−173.39 (19)
C15—Si1—C17—C18	−173.61 (17)	C4—C5—C6—C7	−21.3 (2)
C15—Si1—C17—C19	−53.9 (2)	C5—C6—C7—O7	111.29 (19)
C15—Si1—C17—C20	66.4 (2)	C5—C6—C7—C8	−121.0 (2)
C16—Si1—C17—C18	66.0 (2)	C5—C6—C7—C12	−7.1 (2)
C16—Si1—C17—C19	−174.29 (17)	O7—C7—C8—C9	−157.49 (18)
C16—Si1—C17—C20	−54.1 (2)	C6—C7—C8—C9	73.9 (2)
Si1—O7—C7—C6	60.9 (2)	C12—C7—C8—C9	−36.0 (2)
Si1—O7—C7—C8	−67.9 (2)	O7—C7—C12—C1	43.8 (3)
Si1—O7—C7—C12	172.93 (14)	O7—C7—C12—C4	−86.14 (19)
C11—C1—C2—O2	−81.3 (3)	O7—C7—C12—C10	143.17 (16)
C11—C1—C2—C3	99.2 (2)	C6—C7—C12—C1	163.6 (2)
C12—C1—C2—O2	−177.0 (3)	C6—C7—C12—C4	33.6 (2)
C12—C1—C2—C3	3.5 (3)	C6—C7—C12—C10	−97.06 (18)
C2—C1—C11—C10	−86.9 (2)	C8—C7—C12—C1	−77.8 (3)
C12—C1—C11—C10	15.64 (18)	C8—C7—C12—C4	152.24 (18)
C2—C1—C12—C4	−26.3 (2)	C8—C7—C12—C10	21.5 (2)
C2—C1—C12—C7	−155.6 (2)	C7—C8—C9—C10	35.9 (2)
C2—C1—C12—C10	97.84 (18)	C8—C9—C10—C11	71.4 (2)
C11—C1—C12—C4	−139.21 (19)	C8—C9—C10—C12	−22.4 (2)
C11—C1—C12—C7	91.5 (2)	C8—C9—C10—C14	−159.60 (19)
C11—C1—C12—C10	−15.08 (18)	C9—C10—C11—C1	−120.7 (2)
O2—C2—C3—C4	−159.7 (3)	C12—C10—C11—C1	−14.73 (17)
C1—C2—C3—C4	19.8 (3)	C14—C10—C11—C1	111.5 (2)
C2—C3—C4—C5	−147.8 (2)	C9—C10—C12—C1	130.35 (19)
C2—C3—C4—C12	−34.7 (2)	C9—C10—C12—C4	−119.3 (2)
C2—C3—C4—C13	82.8 (2)	C9—C10—C12—C7	0.6 (2)
C3—C4—C5—C6	155.0 (2)	C11—C10—C12—C1	14.86 (18)
C12—C4—C5—C6	40.8 (2)	C11—C10—C12—C4	125.2 (2)
C13—C4—C5—C6	−77.0 (2)	C11—C10—C12—C7	−114.91 (18)
C3—C4—C12—C1	39.5 (2)	C14—C10—C12—C1	−100.4 (2)
C3—C4—C12—C7	−179.32 (17)	C14—C10—C12—C4	9.9 (3)
C3—C4—C12—C10	−60.5 (2)	C14—C10—C12—C7	129.8 (2)
